# An Alternative Approach to Isoganciclovir: A Prominent Impurity in the Antiviral Drug Ganciclovir

**DOI:** 10.3797/scipharm.1409-15

**Published:** 2014-11-11

**Authors:** Dhanraj T. S. S. Sundaram, Anand G. Kamat, Koilpillai Joseph Prabahar, Peruri Badarinadh Gupta, Battula Venkateswara Rao, Sanasi Paul Douglas

**Affiliations:** 1 Chemical Research and Development, APL Research Centre-II, Aurobindo Pharma Ltd., Survey No. 71 & 72, Indrakaran (V), Medak District-502 329, Andhra Pradesh, India; 2 Department of Engineering Chemistry, Andhra University College of Engineering (A), Andhra University, Visakhapatnam-530003, Andhra Pradesh, India

**Keywords:** Anti-herpes, Cytomegalovirus, Methoxymethyl acetate, Valganciclovir hydrochloride

## Abstract

A simple and efficient process for the preparation of (±)-9-[(2,3-dihydroxypropoxy)methyl]guanine (isoganciclovir) is described. The synthesis features the preparation of a kinetically and thermodynamically controlled acyclic side chain using masked glycerol and methoxymethyl acetate. The unwanted regioisomers were separated through selective crystallization, which upon deprotection yielded isoganciclovir in good yield. The present work explains the preparation of the acyclic side chain in a simple manner without the aid of any preparative column purification or separation technique.

## Introduction

**Fig. 1:**
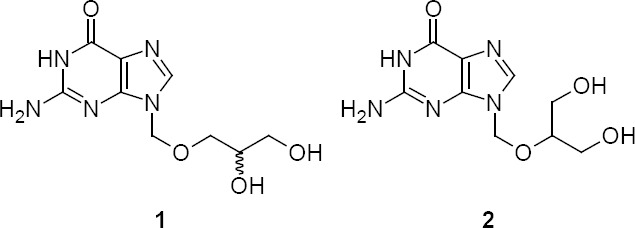
Structures of isoganciclovir (**1**) and ganciclovir (**2**)

Acyclic nucleoside analogues are well-known for their antiviral activities. Some of them are very promising towards the chemotherapy of HSV (herpes simplex virus) infections. Ashton *et al*. were the first to report the anti-herpes properties of (±)-9-[(2,3-dihydroxypropoxy)methyl]guanine **1** (Fig. 1) and its R & S isomers towards HSV type I and type II [1].

**Fig. 2:**
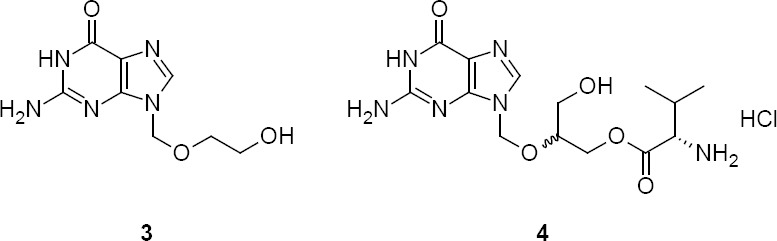
Structures of acyclovir (**3**) and valganciclovir hydrochloride (**4**)

Later works have shown that the S-isomer of **1** is more potent than acyclovir **3** (Fig. 2.), but substantially less effective than ganciclovir **2**. This higher efficacy of **2** is due to its higher degree of solubility in water as well as its bioavailability [2, 3]. Further improvement has been reported where **2** was converted into its prodrug, valganciclovir hydrochloride **4**, an L-valyl ester of ganciclovir that exists as a mixture of two diastereomers. Recently, both of them (**2** and **4**) were approved as effective antiviral drugs for the prevention and treatment of cytomegalovirus (CMV) infections in humans with weak immune systems [4–7].

In general, the synthetic strategy of all acyclic nucleoside analogues involves a convergent synthesis using two synthons such as an acyclic side chain and purine or pyrimidine derivative. In a similar way, the synthesis of the acyclic side chains of **2** and **4** are prepared from a three-carbon synthon such as epichlorohydrin. The undesired ring opening of the epoxide moiety in epichlorohydrin during the preparation of **2** leads to the formation of regioisomers **1**. Such formation of **1** during the manufacturing process of **2** may contaminate the drug substance. The presence of such an impurity in an active pharmaceutical ingredient (API) can have an impact on the quality based on its toxicology. Therefore, it is important to study the formation of such an impurity followed by its control and elimination. The International Conference on Harmonization (ICH) guidelines recommend the identification of such impurities that are present in the API at a level ≥ 0.05% w/w [8]. The pharmacopoeial monograph of ganciclovir specifies the limit of **1** as < 0.5% in US pharmacopoeia and < 0.15% in British and European pharmacopoeia [9–11]. Since **1** is a prominent impurity of ganciclovir, we were interested in exploring a simple and efficient synthetic route for its preparation. Furthermore, the procedures described earlier have certain disadvantages such as (a) a lengthy work-up procedure involving preparative HPLC for the isolation of the acyclic side chain [1], (b) usage of the expensive hydrogenation catalysts for deprotection [13–15], and (c) the reaction with highly corrosive and moisture-sensitive chemicals such as trimethylsilyl chloride [1, 12]. In our present work, we describe a simple approach towards the preparation of **1** without using any harmful or expensive chemicals.

## Results and Discussion

The synthesis of **1** involved two major steps: (i) the preparation of the acyclic side chain and (ii) the condensation of the acyclic side chain with diacetyl guanine.

The route of synthesis of the acyclic side chain is shown in Scheme 1. The selective protection of glycerol was achieved by treating glycerol with cyclohexanone in the presence of a catalytic amount of concentrated sulfuric acid to give ketal **4** [16]. The reaction of **4** with methoxymethyl acetate in the presence of a catalytic amount of *p*-toluenesulfonic acid (*p*-TSA) at room temperature resulted in a mixture of two products. These two products were anticipated in this reaction as 2-[(methoxymethoxy)methyl]-1,4-dioxaspiro[4.5]decane **5** and [(1,4-dioxaspiro[4.5]decan-2-yl)methoxy]methyl acetate **6**. As the product obtained at room temperature was a mixture, the reaction was planned at a lower temperature to get one exclusive product. Surprisingly, at a lower temperature, i.e. -10°C, the only product was **5**. Since the reaction produced exclusively **5** at a lower temperature and a mixture at 25°C, we presumed that **5** may have formed due to the kinetically controlled reaction and **6** could have formed due to the thermodynamically controlled reaction.

**Sch. 1:**
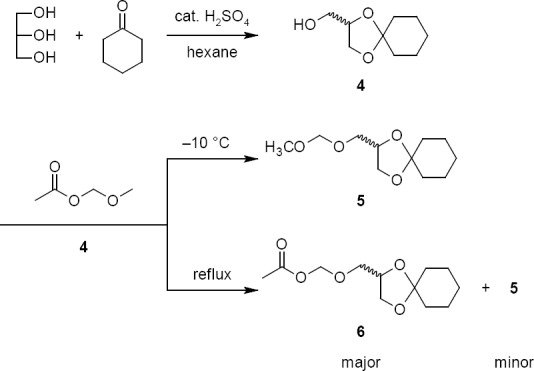
Preparation of acyclic side chains **5** and **6**

**Fig. 3:**
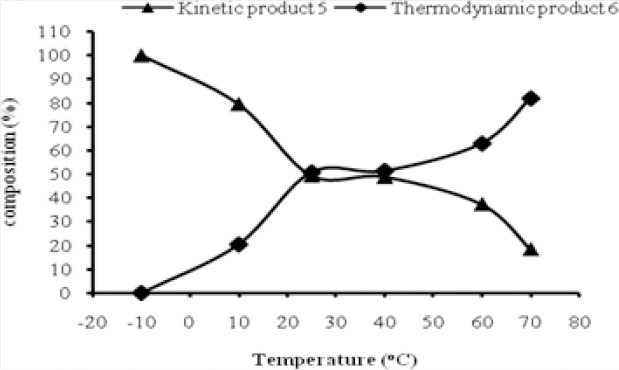
Effect of temperature on the formation of kinetic product **5** and thermodynamic product **6**

To confirm this postulate, the reaction of **4** with methoxymethyl acetate was carried out at different temperatures such as −10°C, 10°C, 25°C, 40°C, 60°C, and at reflux. The products obtained from these reactions were analyzed by ^1^H-NMR to quantify the percentages of **5** and **6** (Fig. 3).

It was clearly seen that the reactions at 25°C, 40°C, 60°C, and at reflux have resulted in nearly 1:1 ratios of **5** and **6**. It was observed that the kinetically controlled product was formed exclusively at low temperature (< −10°C) and converted into the thermosdynamically controlled product at higher temperature. However, repeated experiments have shown that **5** converted into **6** only till the equilibrium was attained and thereafter, further conversion did not take place even upon continuous heating at reflux temperature. This equilibrium was expected due to the retention of the by-product (methanol) in the reaction mixture which prevented the forward reaction. Hence, to facilitate the forward reaction to obtain **6**, the by-product (methanol) had to be distilled out from the reaction mixture. A continuous increase in the percentage of **6** was observed when the reaction was carried out at reflux while distilling the by-product along with the solvent. The product obtained after such an exercise contained the major product **6** with **5** as a minor contaminant. This result has clearly shown an incomplete conversion of **5** into **6**. Among the two leaving groups present in the methoxymethyl acetate, the acetyl group could be protonated easier than the methoxy group and facilitate the formation of kinetic product **5**. We presumed that the thermodynamic product **6** may not have formed directly from the reaction of **4** with methoxymethyl acetate; rather, first the kinetic product forms instantaneously and further, the methoxy group of the kinetic product **5** gets protonated to yield the thermodynamic product **6**. This postulate was confirmed by carrying out a reaction of **4** with methoxymethyl acetate at −10°C and an aliquot was analyzed by ^1^H-NMR to confirm the absence of **6**. Further, the reaction temperature was raised to reflux and the by-product, methanol, was removed simultaneously. The product obtained by this reaction contained ~81.8% of **6** and ~18.2% of **5**. In order to obtain **6** exclusively, the reaction was carried out while adding methoxymethyl acetate at 60°C and at reflux temperature with simultaneous distillation to remove the by-product. The product obtained after 6 h showed ~81.9% of **6** and ~18.1% of **5**. However, prolonged heating has resulted in the degradation with the formation of tarry mass. Further, the reaction was attempted in toluene at reflux temperature where degradation of the product was observed within 1 h, thus failing to give any product. Since the present approach failed to provide the desired results, we changed our strategy by adding a requisite amount of acetic acid into the reaction mixture while removing the by-product to push the reaction in the forward direction. On doing such an exercise, this has resulted in a complete degradation at higher temperature, whereas no reaction at room temperature occurred. Finally, a mixture of **6** and **5** containing ~18.5% of **5** was taken for the next reaction. Both the products **5** and **6** could be used as the acyclic side chain synthons for the preparation of isoganciclovir.

The reaction of acyclic side chain compounds **5** and **6** with diacetyl guanine is shown in Scheme 2. The reaction was started with kinetic product **5** in DMF due to the poor solubility of **7**. The reaction was studied at different conditions and we observed a slower and an incomplete reaction at lower temperature (< 110°C). However, at 110°C, the reaction had taken longer for completion, which upon further optimization, was reduced by adding a catalytic amount of *p*-TSA. As anticipated, the residue obtained after the work-up of the reaction contained a mixture of two regioisomers. These two regioisomers were successfully separated by a selective crystallization technique rather than by column chromatography. The regioisomers were identified as (±)-*N*^2^-acetyl-7-[(1,4-dioxaspiro[4.5]dec-2-yl-methoxy)methyl]guanine **8** and (±)-*N*^2^-acetyl-9-[(1,4-dioxaspiro[4.5]dec-2-yl-methoxy)methyl]guanine **9**, respectively. The required regioisomer **9** was obtained with 32% isolated yield.

**Sch. 2:**
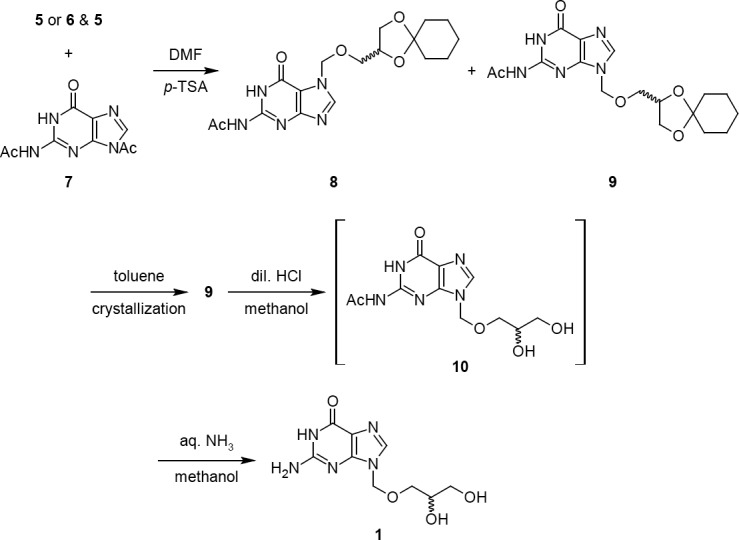
Preparation of isoganciclovir **1**

Similarly, a reaction with thermodynamically controlled product **6** was also performed. Since the thermodynamic reaction yielded predominantly **6** (~81.5%), we have used it as such for the condensation reaction with **7**. We observed a comparatively better yield of **9** (44.2%) after the work-up and crystallization from toluene.

This improvement in the yield was anticipated due to the presence of a better leaving group –O(C)OCH_3_ in **6** rather than –OCH_3_ in **5**. The filtrate mainly contained the *N*^7^-guanine derivative **8** and was isolated and characterized as well. The structures of both regioisomers were confirmed by spectral techniques. These two regioisomers were distinguished by ^1^H-NMR from the downfield shift of *H*-8 (δ 8.36 ppm) and N-C*H*_2_-O (δ 5.68 ppm) protons in **8** with respect to **9**. The slight difference in the downfield chemical shift (~0.20 ppm) of **8** was expected due to the close proximity with the neighbouring carbonyl carbon. These chemical shift values were in agreement with the known *N*^9^ and *N*^7^, *N*^2^-acetyl-(alkoxymethyl)guanines [3]. The deprotection of the cyclohexylidene ring in **9** was carried out with dilute hydrochloric acid in aqueous methanol at 25–30°C to yield *N*^2^-acetyl isoganciclovir **10**. The formation of **10** was confirmed by analysing the reaction mixture by mass spectrometry. The intermediate **10** was not isolated and introduced into the next stage. Finally, the deacetylation of **10** was done in methanol under basic conditions using aqueous ammonia at 25–30°C to obtain the desired **1**.

## Conclusion

A convenient synthesis of **1** was accomplished using acyclic side chain compounds **5** and **6**. The thermodynamic and the kinetic behaviour of **5** and **6** were explained and their impact on the yield was demonstrated.

## Experimental

All reagents were obtained commercially and used as received unless otherwise stated. Solvents were dried as per the standard procedures. Melting points were determined on the Reichert thermopan melting point apparatus. The ^1^H- and ^13^C-NMR spectra were recorded on the Bruker Avance 300 MHz and Varian 500 MHz spectrometers using TMS as the internal standard in DMSO-d_6_ and CDCl_3_. The FTIR spectra were recorded on a Perkin-Elmer Spectrum One Fourier transform FTIR spectrophotometer. High-resolution mass spectral analyses were performed using the electrospray ionization (ESI) method on the Xevo G2 QTOf mass spectrometer. HPLC analyses were carried out as described in the European Pharmacopoeia for ganciclovir related substances.

### 2-[(Methoxymethoxy)methyl]-1,4-dioxaspiro[4.5]decane (5)

2-Hydroxymethyl-1,4-dioxaspiro[4.5]decane (**4**) (100.0 g, 0.58 mol), *p*-TSA.H_2_O (2.0 g) and methoxymethyl acetate (302.3 g, 2.90 mol) were taken in hexane (500 mL) at −10°C and the reaction mixture was stirred for 2 h at −10°C. After the completion, the reaction mixture was washed with a saturated solution of aqueous sodium carbonate (2 x 100 mL) and saturated aqueous sodium chloride solution (100 mL). The organic layer was concentrated at 35–40°C under reduced pressure to afford **5** as viscous brown oil. Yield: 98.6 g (78.5%); ^1^H-NMR (300 MHz, CDCl_3_): δ 1.37–1.41 (m, 2H), 1.56–1.67 (m, 8H), 3.36 (s, 3H), 3.54–3.63 (m, 2H), 3.72-3.77 (dd, *J1* = 6.0 Hz and *J2* = 6.0 Hz, 1H), 4.04–4.09 (dd, *J1* = 6.3 Hz and *J2* = 6.3 Hz, 1H), 4.27–4.32 (m, 1H), 4.66 (s, 2H); ^13^C-NMR (DEPT, 125 MHz, DMSO-*d*_6_): δ 110.0, 96.5, 74.2, 68.7, 66.3, 55.1, 36.4, 34.8, 25.1, 23.9, 23.7; FTIR (neat) cm^−1^: 3054, 2862, 1670, 1607; HR-MS (ESI, QTOF) for C_11_H_20_O_4_Na [M + Na]^+^: *m/z* calcd: 239.1259; found: 239.1265.

### (1,4-Dioxaspiro[4.5]decan-2-ylmethoxy)methyl acetate (6)

2-Hydroxymethyl-1,4-dioxaspiro[4.5]decane (**4**) (60.0 g, 0.35 mol) was dissolved in hexane (120 mL) and heated to 60°C under a nitrogen atmosphere. Thereafter, *p*-TSA.H_2_O (2.0 g) and methoxymethyl acetate (180.0 g, 1.73 mol) were added at 60°C. The reaction mixture was refluxed for 6 h and methanol was removed by distillation during the reaction. The reaction volume was maintained by adding fresh hexane to the reaction mixture. After the completion of the reaction, the reaction mixture was washed with 5% w/v aqueous sodium carbonate solution (120 mL) and the organic layer was separated. The aqueous layer was extracted with hexane (60 mL) and further, the combined organic layer was washed with 5% w/v aqueous sodium carbonate solution (60 mL). The organic layer was concentrated at 35–40°C to afford **6** as viscous pale yellow oil. Yield: 58.8 g (containing ~18.5% of **5**); ^1^H-NMR (300 MHz, CDCl_3_): δ 1.37–1.47 (m, 2H), 1.59–1.62 (m, 8H), 2.10 (s, 3H), 3.66–3.76 (m, 3H), 4.02–4.07 (m, 1H), 4.27 (m, 1H), 5.30 (s, 2H); ^13^C-NMR (DEPT, 125 MHz, DMSO-*d*_6_): δ 170.4, 109.9, 89.2, 73.9, 71.1, 66.1, 36.4, 34.8, 25.1, 23.8, 23.7, 20.9; FTIR (neat) cm^−1^: 2936, 2862, 1748; HR-MS (ESI, QTOF) for C_12_H_20_O_5_Na [M + Na]^+^: *m/z* calcd: 267.1209; found: 267.1207.

### (±)-N^2^-acetyl-9-[(1,4-dioxaspiro[4.5]decan-2-yl-methoxy)methyl]guanine (9)

To a stirred suspension of diacetyl guanine **7** (30.0 g, 0.13 mol) in dimethylformamide (150 mL), [(1,4-dioxaspiro[4.5]decan-2-yl)methoxy]methyl acetate (**6)** (62.3 g, 0.25 mol, containing ~18.5% of **5**) and *p*-TSA.H_2_O (1.20 g) were added. The reaction mixture was stirred at 110°C for 6 h. Thereafter, the reaction mixture was cooled to 25–30°C and water (150 mL) was added. The product was extracted with ethyl acetate (2 x 150 mL). The organic layer was washed with 20% w/w brine (100 mL) and concentrated at 45–50°C under reduced pressure. The residue was dissolved in toluene (120 mL) at 55–60°C and cooled to 25–30°C. The precipitated mass was filtered and washed with ice-cold isopropyl alcohol (30 mL) to obtain **9** as an off-white solid. Yield: 21.3 g (44.2%); Mp: 186–188°C; ^1^H-NMR (300 MHz, DMSO-*d_6_*): δ 1.25–1.33 (m, 2H), 1.37–1.57 (m, 8H), 2.18 (s, 3H), 3.51–3.55 (m, 3H), 3.89–3.95 (dd, *J1* = 6.5 Hz and *J2* = 8.1 Hz, 1H), 4.10–4.14 (m, 1H), 5.48 (s, 2H), 8.14 (s, 1H), 11.78 (s, 1H), 12.07 (s, 1H); ^13^C-NMR (PENDANT, 75 MHz, DMSO-*d*_6_): δ 173.5, 154.8, 148.8, 148.0, 140.0, 120.2, 109.0, 73.5, 72.8, 69.6, 65.4, 35.9, 34.4, 24.6, 23.7, 23.5, 23.4; FTIR (KBr) cm^−1^: 3239, 3168, 2935, 1691, 1607; HR-MS (ESI, QTOF) for C_17_H_23_N_5_O_5_ [M+H]^+^: *m/z* calcd: 378.1778; found: 378.1777.

### (±)-N^2^-acetyl-7-[(1,4-dioxaspiro[4.5]decan-2-yl-methoxy)methyl]guanine (8)

The N^7^-isomer was isolated from the filtrate, containing ~20% of **9** (slightly lower R*_f_* on TLC; CHCl_3_-MeOH, 4:1). ^1^H-NMR (300 MHz, DMSO-*d*_6_): δ 1.28–1.32 (m, 2H), 1.37–1.56 (m, 8H), 2.18 (s, 3H), 3.47–3.55 (m, 3H), 3.90–3.94 (dd, *J1* = 6.5 Hz and *J2* = 8.1 Hz, 1H), 4.10–4.12 (m, 1H), 5.68 (s, 2H), 8.37 (s, 1H), 11.62 (br s, 1H), 12.16 (br s, 1H); ^13^C-NMR (PENDANT, 75 MHz, DMSO-*d*_6_): δ 173.4, 157.5, 152.4, 147.2, 145.0, 111.0, 109.0, 75.3, 73.6, 69.5, 65.4, 35.9, 34.5, 24.6, 23.7, 23.5, 23.4; HR-MS (ESI, QTOF) for C_17_H_23_N_5_O_5_ [M + H]^+^: *m/z* calcd: 378.1778; found: 378.1780.

### (±)-9-[(2,3-dihydroxypropoxy)methyl]guanine (1)

To a stirred solution of **9** (5.0 g, 0.01 mol) in methanol (25 mL), dilute hydrochloric acid (10% w/w, 15 mL) was added at 25–30°C. The reaction mixture was stirred for 3 h, thereafter concentrated under reduced pressure at 45–50°C. The residue contained the intermediate **10**, which was further stirred into a mixture of aqueous ammonia (15 mL) and methanol (20 mL) for 20 h at 25–30°C. The suspension was concentrated under reduced pressure and crystallized from aqueous methanol to get **1** as an off-white solid. Yield: 3.1 g (91.7%); Purity by HPLC: 93.95%; Mp: 242–246°C; ^1^H-NMR (300 MHz, DMSO-*d*_6_): δ 3.26–3.32 (t, *J* = 5.7 Hz, 2H), 3.44–3.49 (m, 2H), 4.49-4.53 (t, *J* = 5.7 Hz, 1H), 4.74 (d, *J* = 4.8 Hz, 1H), 5.34 (s, 2H), 6.50 (br s, 2H), 7.81 (s, 1H), 10.63 (s, 1H); ^13^C-NMR (PENDANT, 75 MHz, DMSO-*d*_6_): δ 156.9, 153.8, 151.4, 137.9, 116.4, 72.3, 70.8, 70.4, 62.8; FTIR (KBr) cm^−1^: 3387, 3157, 2933, 2881, 1694; HR-MS (ESI, QTOF) for C_9_H_13_N_5_O_4_ [M + H]^+^: *m/z* calcd: 256.1047; found: 256.1054.
